# Novel diagnostic and prognostic approach for rapidly progressive dementias: Indicators based on amyloid/tau/neurodegeneration (ATN) framework

**DOI:** 10.1111/cns.14857

**Published:** 2024-07-16

**Authors:** Yuan Cheng, Shu‐Fen Chen, Ya‐Ru Zhang, Yu Guo, Kai‐Min Wu, Yu‐Yuan Huang, Qiaolifan Aerqin, Kevin Kuo, Hong‐Qi Li, Shi‐Dong Chen, Wei‐Shi Liu, Qiang Dong, Jin‐Tai Yu

**Affiliations:** ^1^ State Key Laboratory of Medical Neurobiology and MOE Frontiers Center for Brain Science, Department of Neurology and Institute of Neurology, Huashan Hospital, Shanghai Medical College Fudan University Shanghai China; ^2^ National Center for Neurological Disorders Shanghai China

**Keywords:** ATN framework, autoimmune encephalitis, Creutzfeldt–Jakob disease, diagnosis, prognosis, rapidly progressive Alzheimer's disease

## Abstract

**Aims:**

Apply established cerebrospinal fluid (CSF) and serum biomarkers and novel combined indicators based on the amyloid/tau/neurodegeneration (ATN) framework to improve diagnostic and prognostic power in patients with rapidly progressive dementias (RPDs).

**Methods:**

CSF and serum biomarkers of Alzheimer's disease (AD) common neuropathology including Aβ42, Aβ40, p‐Tau, and t‐Tau were measured in cognitively normal (CN) controls (*n* = 33) and three RPD groups with rapidly progressive AD (rpAD, *n* = 23), autoimmune encephalitis (AE, *n* = 25), and Creutzfeldt–Jakob disease (CJD, *n* = 28). Logistic regression and multiple linear regression were used for producing combined indicators and prognostic assessment, respectively, including *A&T, A&N, T&N, A&T&N*, etc.

**Results:**

Combined diagnostic indicator with *A&T&N* had the potential for differentiating AE from other types of RPDs, identifying 62.51% and 75% of AE subjects based on CSF and serum samples, respectively, compared to 39.13% and 37.5% when using autoantibodies. CSF t‐Tau was associated with survival in the CJD group (adjusted *R*‐Square = 0.16, *p* = 0.02), and its prognosis value improved when using combined predictors based on the ATN framework (adjusted *R*‐Square = 0.273, *p* = 0.014).

**Conclusion:**

Combined indicators based on the ATN framework provide a novel perspective for establishing biomarkers for early recognition of RPDs due to treatment‐responsive causes.

## Graphical abstract

The current study demonstrated for the first time that: (1) Indicators based on the ATN framework using CSF biomarkers had better diagnostic and prognostic power for RPDs, especially for autoantibodies‐negative AE subjects. (2) Indicators based on the ATN framework using serum biomarkers still showed diagnostic value, which provides efficient clinical information for early recognition of treatment‐responsive causes of RPDs.

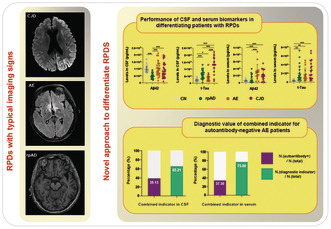

## 1 INTRODUCTION

Rapidly progressive dementias (RPDs) are cognitive disorders with fast progression, which develop more commonly over weeks to months.^1^ RPDs have various etiology and prognosis. Compared to most slowly progressive dementias, some types of RPDs can be quickly fatal.^2^ One of the prototypical causes of RPDs, Creutzfeldt–Jakob disease (CJD) caused by a type of misfolded prion (PrP^Sc^), always inevitably results in death after a very short duration.^3^ Apart from CJD, the most common causes of RPDs are atypical manifestations of neurodegenerative diseases containing rapidly progressive Alzheimer's disease (rpAD), as well as curable disorders such as autoimmune encephalopathies (AE). RPDs due to reversible causes should be identified early as some types of RPDs are clinically intervenable. Hence, accurate and prompt differential diagnosis is critical.

RPDs due to AE can always be treatable, unfortunately, diagnosis of antibody‐negative AE is clinically challenging. In the absence of autoantibodies, the diagnosis of AE is often hindered by the lack of specificity of the most frequent presenting symptoms and signs.^4^ CJD and rpAD sometimes mimic the symptoms of AE, which makes early clinical treatment difficult. Therefore, there is an urgent need to establish new diagnostic methods for AE, especially antibody‐negative AE. It is also critical to identify fatal prion diseases as soon as possible. For a long time, the diagnosis of CJD has been based on criteria that consider EEG, MRI, CSF 14‐3‐3 proteins, and PrP^Sc^ detected by real‐time quaking‐induced conversion (RT‐QuIC) as biomarkers to support a probable clinical diagnosis.^5^, ^6^, ^7^, ^8^ However, RT‐QuIC is not fully commercialized and can only be tested in certain qualified institutions in some regions. Elevated amounts of 14‐3‐3 protein in CSF are usually a common indicator of CJD, while 14‐3‐3 protein in serum lacks the same diagnostic power. Recently, some CSF biomarkers including NFL, GFAP, SNAP‐25, MCP‐1, and sTREM2 have been shown to have the potential to differentiate certain types of RPDs,^9^ while efficient and simple diagnostic tools are still lacking.

Aβ was once considered a unique pathological substance of AD, but plenty of studies have shown that Aβ pathology also occurs in some other dementia types.^10^ Tau pathology, as a marker of neuronal damage, can also appear in a variety of rapidly progressive neurological diseases. This suggests that Aβ and Tau may exist in a common pathway in rapidly progressive dementia. Indeed, CSF biomarkers including Aβ and Tau have established utility to discriminate patients with RPDs due to CJD from other types of RPDs.^11^, ^12^ Moreover, CSF markers of neurodegeneration, such as total‐Tau (t‐Tau) are even used for disease prognosis in CJD patients.^13^ Altogether, this implies that the combination of indicators based on amyloid/tau/neurodegeneration (ATN) framework may have potential in diagnosis and prognosis for RPDs. However, whether indicators or combinatorial biomarker panels based on the ATN framework have diagnostic and prognostic power for AE, rpAD, and CJD in the context of RPDs remains unclear.

The current study first investigated the performance of fluid biomarkers and indicators based on the ATN framework in differentiating RPDs, especially in identifying autoantibodies‐negative AE subjects. Further, we explored the associations of fluid biomarkers with cognitive decline in rpAD and AE individuals, survival time in CJD subjects, and the prognostic power of indicators based on the ATN framework for RPDs.

## 2 METHODS

### 2.1 Study population

All participants in this study were recruited from Huashan Hospital between November 2019 and October 2022. Individuals were excluded for the following reasons: (1) concomitant neurologic disorder (multiple sclerosis, Parkinson's disease, epilepsy, etc.); (2) severe systemic diseases (liver insufficiency, renal insufficiency, cancer, special infections, etc.); (3) enduring mental illness (e.g., schizophrenia); (4) people who are reluctant to lumbar puncture or participate in this study. The current study was approved by the Institutional Review Board of Huashan Hospital, and all participants and their caregivers provided informed consent.

### 2.2 Clinical assessments and diagnosis of CJD, AE, and rpAD

The clinical assessments of CJD, AE, and rpAD were conducted following the previously used protocol.^14^, ^15^, ^16^ Briefly, the demographic characteristics (including age, sex, and education level), and comorbidities (including diabetes, hypertension, coronary heart disease, dyslipidemia, etc.) were collected. Then, the common clinical assessments were undergone including physical examination, laboratory tests, magnetic resonance imaging (MRI), electroencephalogram (EEG), neuropsychological tests, CSF autoimmune antibody, and AD biomarkers assays.

Diagnosis of CJD in the current study was based on the most recently updated diagnostic criteria for CJD and related disorders.^8^ In all 28 CJD cases enrolled in the current study, two individuals carried *PRNP* variants (c.695 T>G and c.572 C>T, respectively) and were considered hereditary CJD, and the remaining 26 CJD patients were probable CJD fulfilling the clinical criteria for possible CJD and showing either periodic sharp wave complexes (PSWCs) on the EEG or specific diffusion‐weighted imaging (DWI)‐MRI scan results. In total, there were 12 individuals with EEG characteristic changes, 25 individuals with specific DWI‐MRI scan results, and nine individuals with both characteristic EEG and DWI‐MRI scan results.

Diagnosis of AE was based on the routine procedures established previously.^17^ Patients were enrolled if they met diagnostic criteria for definite AE or probable AE with antibody‐negative. The current study contains nine definite AE patients, of which, five individuals with anti‐Leucine‐rich glioma‐inactivated‐1 (LGI‐1) antibody, two individuals with anti‐N‐methyl‐D‐aspartate (NMDA) antibody, 1 individual with anti‐alpha‐amino‐3‐hydroxy‐5 ‐methyl‐4‐isoxazole propionic acid‐2 (AMPA‐2) antibody, one individual with anti‐paraneoplastic antigen Ma‐2 antibody. The remaining 14 individuals were defined as probable AE with antibody‐negative.

Diagnosis of AD dementia was made according to the criteria of the National Institute of Neurological and Communicative Disorders and Stroke and the Alzheimer's Disease and Related Disorder Association (NINCDS‐ADRDA).^18^ Both CSF‐AD biomarkers and the core clinical criteria were used for diagnosis. A general but clinically valuable definition of rpAD was used in the current study, which refers to an obvious deterioration in the cognitive status of AD dementia patients within a short period (no more than 12 months).

### 2.3 CSF and serum samples collection and AD biomarkers assessment

CSF samples were collected by lumbar puncture and processed according to a standard procedure. Specifically, the CSF samples without visible blood contamination were centrifuged at 2000 × *g* at room temperature for 10 min, and the aliquots were then immediately frozen and stored at −80°C until use. CSF levels of A42, Aβ40, t‐Tau, and phosphorylated tau‐181 (p‐Tau) were measured using human Aβ and Tau ELISA kits (Innotest, United States). Fasting blood was collected between 07:00 and 09:00. The blood samples were centrifuged within an hour of collection, and serum was aliquoted into 0.5 mL polypropylene tubes and stored at −80°C until use. All the serum samples were measured by single molecular immunity detection (SMID; Suzhou AstraBio Technology Co., Ltd, China). Informed consent was obtained before the acquisition of the CSF and blood samples. All measurements were performed by experienced laboratory technicians in a blinded approach.

### 2.4 Cognitive measures at baseline and follow‐up

Cognitive measures were conducted as the previous study described.^19^ Briefly, at baseline, the cognitive and functional status of participants with memory and cognitive complaints was assessed using a neuropsychological battery that included the Mini‐Mental State Examination (MMSE), Montreal Cognitive Assessment (MoCA), activities of daily living (ADL), clinical dementia rating (CDR), Hamilton Anxiety Rating Scale (HAM‐A), Hamilton Depression Rating Scale (HDRS), and Pittsburgh sleep quality index (PSQI). In follow‐up, telephone interviews were conducted to assess cognition with the professionally trained neurologist. Current cognitive status was assessed using the Chinese version of the Telephone Interview of Cognitive Status‐40 (TICS‐40),^20^ which includes 10 variables and has a maximum of 40 points. Based on a previously reported method,^21^ we converted TICS‐40 scores to MMSE scores and defined the cognitive decline rate as the ratio of MMSE scores changes to follow‐up time.^21^

### 2.5 Statistical analyses

Demographic characteristics with continuous variables are described as the median/mean, and categorical data are summarized as absolute frequencies. One‐way ANOVA (followed by Tukey's post hoc test) was applied for multiple group comparisons. The Bonferroni correction was applied to analysis with multiple comparisons. Receiver operating characteristic (ROC) curve analyses were carried out to establish the diagnostic sensitivity and specificity of each biomarker or combination of biomarkers, and areas under the curve (AUC) with 95% confidence intervals (95% CI) were calculated. Comparison among different AUCs was performed via the DeLong Test using MedCalc software (Version 22.023). The optimal cut‐off values used for the identification of each positive status (A+, T+, N+) according to ATN classification were estimated based on the maximized Youden index. Specifically, logistic regression using SPSS software produces predicted values, which can be used to draw a ROC curve for comparison between the two groups. The cut‐off value represents the predicted value when the Yuden index is maximum. Cut‐off values using CSF and serum samples for distinguishing rpAD versus AE were Aβ42 (560.70, 10.06), Aβ42/Aβ40 (0.095, 0.048), p‐Tau (53.80, 9.70), t‐Tau (695.80, 69.69), respectively. Cut‐off values used for distinguishing rpAD versus CJD were Aβ42 (574.50, 9.10), Aβ42/Aβ40 (0.095, 0.020), p‐Tau (73.98, 9.30), t‐Tau (1070.00, 151.20), respectively. Cut‐off values used for distinguishing AE versus CJD were Aβ42 (685.40, 18.57), Aβ42/Aβ40 (0.183, 0.026), p‐Tau (34.48, 11.40), and t‐Tau (752.30, 153.20), respectively. The combined diagnostic indicators of the ATN framework were established by logistic regression (forward method), with Aβ42 or Aβ42/40 (A), p‐Tau181 (T), and t‐Tau (N) as independent variables. Moreover, demographic information, including age, sex, follow‐up time, and disease duration, was added incrementally to the optimal model by logistic regression (forward method). The partial correlation analysis was used to detect the strength of the correlation between some of the variables. Logistic regression was used to analyze the predictive effect of the CSF biomarkers for survival outcomes, and multiple linear regression was applied to analyze the prognostic value of the biomarkers on cognitive decline rate.

Statistical analysis was performed using IBM SPSS Statistics version 22 software (IBM, Armonk, NY, USA). All figures were completed using GraphPad Prism version 8.0 software (San Diego, USA). All hypothesis testing was two‐sided, and *p* < 0.05 was defined as statistically significant.

## 3 RESULTS

### 3.1 Characteristics of the participants and comparison of biomarkers and their ratios in patients with RPDs

The characteristics of the subjects are shown in Table 1. The study consisted of 25 rpAD patients, 23 AE patients, 28 CJD patients, and 33 cognitively normal (CN) controls. All the participants have CSF samples, yet partial subjects have serum samples (*n* = 21 in rpAD and CJD groups, *n* = 16 in AE group, and *n* = 33 in CN controls). There were no significant differences in age, sex, education level, or comorbidities (diabetes mellitus, hypertension, cardiovascular disease, and dyslipidemia) among these groups. The MMSE score and duration of follow‐up time (survival time in CJD patients) were significantly lower in the CJD group than those in the rpAD and AE groups (*p* < 0.0001).

**TABLE 1:** Demographics data of the CN controls and subjects in patients with RPDs.

Characteristics	CN (*n* = 33)	Subjects with CSF samples				Subjects with serum samples			
		rpAD (*n* = 25)	AE (*n* = 23)	CJD (*n* = 28)	*p* value	rpAD (*n* = 21)	AE (*n* = 16)	CJD (*n* = 21)	*p* value
Age, mean (SD), years	57.82 (7.02)	61.60 (9.58)	57.13 (12.78)	60.93 (8.98)	0.2867	61.33 (8.71)	60.31 (10.45)	62.67 (8.01)	0.7273
Female, *N* (%)	18 (54.55)	18 (72.00)	11 (47.83)	19 (67.86)	0.1800	16 (76.19)	7 (43.75)	12 (57.14)	0.0880
Education, median (SD), years	13.38 (2.44)	9 (4.24)	10.57 (3.25)	10.82 (3.50)	0.1687	8.86 (3.85)	10.50 (3.46)	10.57 (3.50)	0.2438
MMSE (at baseline), median (SD)	28.97 (1.19)	15.96 (6.28)	11.65 (9.96)	10.71 (8.71)	0.0655	15.33 (7.00)	14.56 (9.80)	10.52 (2.06)	0.0577
MMSE (at follow‐up time), median (SD)	/	12.20 (6.60)^c^	11.22 (8.91)^c^	3.67 (5.02)^a^, ^b^	<0.0001	11.62 (6.59)^c^	14.19 (8.70)^c^	3.57 (3.67)^a^, ^b^	<0.0001
Duration of follow‐up (survival time in CJD group), mean (SD), months	/	12.72 (6.12)^c^	12.87 (6.31)^c^	5.77 (5.67)^a^, ^b^	<0.0001	13.19 (6.56)^c^	14.44 (6.97)^c^	5.64 (6.03)^a^, ^b^	<0.0001
Comorbidities, *n* (%)									
Diabetes	17 (51.52)	5 (20.00)	4 (17.39)	4 (14.29)	0.9781	4 (19.05)	3 (18.75)	4 (19.05)	0.9997
Hypertension	12 (36.36)	9 (36.00)	10 (43.48)	10 (35.71)	0.8623	7 (33.33)	6 (36.50)	8 (38.10)	0.9422
Coronary artery disease	8 (24.24)	6 (24.00)	6 (26.09)	7 (25.00)	0.9621	5 (23.81)	3 (18.75)	5 (23.81)	0.9183
Dyslipidemia	7 (21.21)	8 (32.00)	4 (17.39)	7 (25.00)	0.9633	6 (28.57)	3 (18.75)	6 (28.57)	0.7472

*Note*: Results are shown as mean (SD) or number (%).

Abbreviations: AE, autoimmune encephalitis; CJD, Creutzfeldt–Jakob disease; CN, cognitively normal; MMSE, Mini‐Mental State Examination; *N*, number; rpAD, rapidly progressive Alzheimer's disease; SD, standard deviations. MMSE (at follow‐up time): *p* value (CSF rpAD vs CJD) = 3.1e(−5), *p* value (CSF AE vs CJD) = 2.56e(−5), *p* value (serum rpAD vs CJD) = 5.800058e(−5), *p* value (serum AE vs CJD) = 0.7e(−5). Duration of follow‐up: *p* value (CSF rpAD vs CJD) = 7.4e(−5), *p* value (CSF AE vs CJD) = 7.5e(−5), *p* value (serum rpAD vs CJD) = 4.03e(−5), *p* value (serum AE vs CJD) = 1.45e(−5).

^a^ *p* < 0.05 compared to the rpAD group.

^b^ *p* < 0.05 compared to the AE group.

^c^ *p* < 0.05 compared to the CJD group.

**FIGURE 1:**
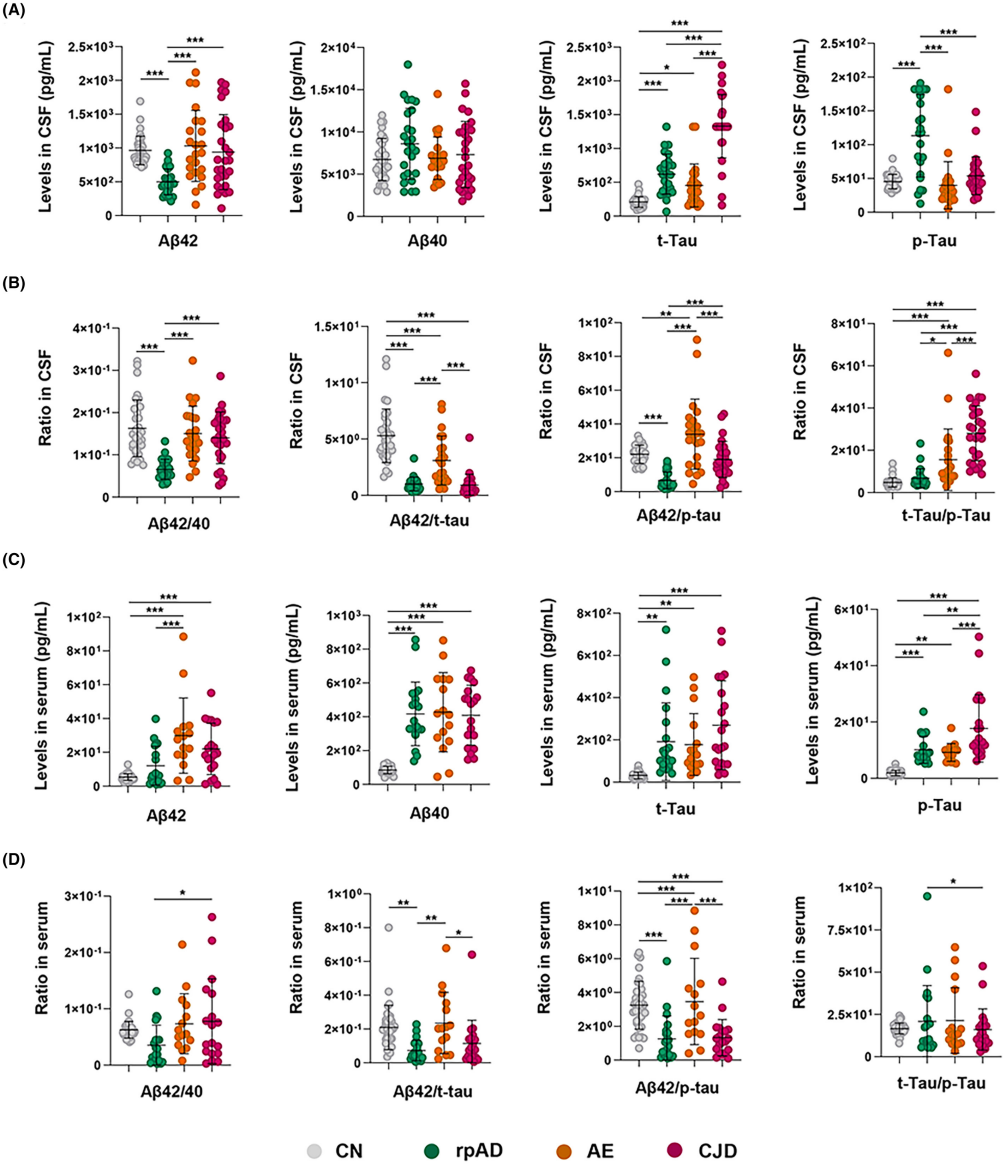
Levels of CSF and serum biomarkers in patients with RPDs. (A) Comparison of CSF biomarkers levels among CN, rpAD, AE, and CJD subjects. (B) Comparison of CSF biomarkers ratios among CN, rpAD, AE, and CJD subjects. (C) Comparison of serum biomarkers levels among rpAD, AE, and CJD patients. (D) Comparison of serum biomarkers ratios among CN, rpAD, AE, and CJD subjects. One‐Way ANOVA Test. *P* values are indicated with asterisks: **p < 0.01, ***p < 0.001. AE, autoimmune encephalitis; Aβ, β‐amyloid; CJD, Creutzfeldt–Jakob disease; CN, cognitively normal; CSF, cerebrospinal fluid; p‐Tau, phosphorylated Tau protein; rpAD, rapidly progressive Alzheimer's disease; t‐Tau, total Tau protein.

The comparison of CSF and serum AD biomarkers in these groups is shown in Figure 1A,C. Specifically, the CSF Aβ42 levels in rpAD group were lower than that of the AE (*p* < 0.001) and CJD (*p* < 0.001) groups, as well as CN subjects (*p* < 0.001). The serum Aβ42 levels were all elevated in patients with RPDs compared to the CN group, and in the rpAD group, serum Aβ42 levels were also lower than that of the AE group (*p* < 0.001). In addition, the CSF p‐Tau levels in the rpAD group were higher than that of the AE, CJD, and CN groups (*p* < 0.001), while the serum p‐Tau levels in the CJD group were higher than that of the other three groups (*p* < 0.01). Besides, the CSF t‐Tau levels in the CJD group were significantly greater than that of the rpAD, AE, and CN groups (*p* < 0.001), yet the serum t‐Tau levels did not differ among these three groups, nevertheless, serum t‐Tau levels in patients with RPDs were higher than the CN group. In the meanwhile, the ratios of CSF and serum AD biomarkers were also compared among the four groups (Figure 1B,D). It is worth mentioning that Aβ42/t‐Tau and Aβ42/p‐Tau in the AE group were higher than that of the other RPD groups in both CSF and serum samples (*p* < 0.05).

### 3.2 Performance of CSF and serum biomarkers in differentiating patients with RPDs

CSF Aβ42 was able to differentiate rpAD from AE and CJD groups with moderate accuracy (AUC = 0.8348 and 0.7543, respectively), while serum Aβ42 can only distinguish rpAD from AE (AUC = 0.7778). Neither CSF nor serum Aβ40 could differentiate the three diseases. By contrast, the t‐Tau and p‐Tau in CSF can discriminate between any two diseases. The serum p‐Tau can discriminate CJD from rpAD and AE with moderate accuracy (AUC = 0.7744 and 0.7902, respectively), while serum t‐Tau cannot discern among these three disorders. Moreover, the diagnostic power of biomarker ratios was also evaluated. Aβ42/p‐Tau in both CSF and serum showed better accuracy (AUC = 0.9443 and 0.8159, respectively) to distinguish rpAD from AE group, and Aβ42/t‐Tau in both CSF and serum were able to distinguish AE from rpAD and CJD groups. The CSF t‐Tau/p‐Tau was able to distinguish among these three disorders, while the t‐Tau/p‐Tau in serum could not discriminate between any two diseases (Tables S1 and S2).

### 3.3 Performance of indicators based on ATN framework for differentiating patients with RPDs

To improve the diagnosis accuracy, novel indicators based on the ATN framework were established to differentiate patients with rpAD, AE, and CJD, based on CSF and serum biomarkers and demographic characteristics (age, disease duration), including *A&T, A&N, T&N, A&T&N*, etc, by logistic regression (Tables 2 and 3).

**TABLE 2:** Performance of indicators based on ATN framework using CSF biomarkers for differentiating patients with RPDs.

Indicators	A & T		A & N		T & N		A & T & N	
	Cutoff	AUC (95% CI)	Cutoff	AUC (95% CI)	Cutoff	AUC (95% CI)	Cutoff	AUC (95% CI)
rpAD versus AE	>0.6350	0.9583* (0.9092–1.0000)	>0.6343	0.9652* (0.9221–1.000)	>0.595	0.8348* (0.7106–0.9590)	>0.6343	0.9652* (0.9221–1.000)
CJD versus rpAD	>0.7508	0.9057* (0.8300–0.9814)	>0.6338	0.9371* (0.8609–1.000)	>0.340	0.9786* (0.9487–1.000)	>0.3569	0.9857* (0.9605–1.000)
CJD versus AE	>0.4868	0.5611* (0.5657–0.8567)	>0.4554	0.9193* (0.8355–1.000)	>0.4629	0.9224* (0.8402–1.000)	>0.4745	0.9224* (0.8386–1.000)

Indictors	AN & age		ATN & age		TN &duration	
	Cutoff	AUC (95% CI)	Cutoff	AUC (95% CI)	Cutoff	AUC (95% CI)
rpAD versus AE	>0.6144	0.9791* (0.9485–1.000)	/	/	/	/
CJD versus rpAD	/	/	>0.8336	0.9943* (0.9823–1.000)	/	/
CJD versus AE	/	/	/	/	>0.5988	0.9239* (0.8524–0.9954)

*Note*: *p* values are indicated with asterisks: *p* < 0.05.

Abbreviations: AE, autoimmune encephalitis; AN, Amyloid/Neurodegeneration; AT, amyloid/tau; ATN, amyloid/tau/neurodegeneration; rpAD, rapidly progressive Alzheimer's disease; TN, tau/neurodegeneration.

**TABLE 3:** Performance of indicators based on ATN framework using serum biomarkers for differentiating patients with RPDs.

Indicators	A & T		A & N		T & N		A & T & N	
	Cutoff	AUC (95% CI)	Cutoff	AUC (95% CI)	Cutoff	AUC (95% CI)	Cutoff	AUC (95% CI)
rpAD versus AE	>0.363	0.7738* (0.6167–0.9309	>0.352	0.7619* (0.5996–0.9242)	<0.444	0.5089* (0.3147–0.7032)	>0.384	0.7946* (0.6423–0.9470)
CJD versus rpAD	>0.445	0.7494* (0.6006–0.8982))	>0.426	0.6497 (0.4794–0.8199)	>0.385	0.7721* (0.6287–0.9155)	>0.458	0.7494* (0.6007–0.8982)
CJD versus AE	>0.442	0.8378* (0.7062–0.9694)	>0.466	0.7574* (0.5923–0.9226)	>0.558	0.7872* (0.6407–0.9337)	>0.404	0.8527* (0.7302–0.9751)

Indicators	AT & age		ATN & age		TN &duration	
	Cutoff	AUC (95% CI)	Cutoff	AUC (95% CI)	Cutoff	AUC (95% CI)
rpAD versus AE			>0.4996	0.8333* (0.7012–0.9655)		
CJD versus rpAD					>0.4414	0.8549* (0.7422–0.9676)
CJD versus AE			>0.7754	0.8810* (0.7756–0.9863)		

*Note*: *p* values are indicated with asterisks: *p* < 0.05.

Abbreviations: AE, autoimmune encephalitis; AN, amyloid/neurodegeneration; AT, amyloid/tau; ATN, amyloid/tau/neurodegeneration; rpAD, rapidly progressive Alzheimer's disease; TN, tau/neurodegeneration.

The combined indicator including *A&T* in CSF showed potential for differentiating between rpAD and AE subjects (AUC = 0.9583), moreover, the CSF indicator containing *AN & age* performed better with higher accuracy (AUC = 0.9791). In addition, the combined indicator of CSF *ATN & age*, and CSF *TN & duration*, performed great in the discrimination of CJD and rpAD subjects (AUC = 0.9943), as well as CJD and AE subjects (AUC = 0.9239), respectively. Besides, the indicator based on the ATN framework in serum also had the potential to distinguish these diseases (Table 3). Especially, the indicator of *ATN & age* showed potential for differentiating AE from rpAD and CJD subjects (AUC = 0.8333 and 0.8810, respectively). However, all the AUCs of combined indicators using serum biomarkers had a certain decline compared with those using CSF biomarkers. In addition, the current study includes CSF Aβ42 and Aβ42/40, respectively, to reflect amyloid pathology based on the ATN framework in diagnostic analysis and found that the maximum AUCs for rpAD versus AE, as well as rpAD versus CJD were all elevated when using Aβ42/40 as an ATN component. Only the maximum AUCs were presented here.

### 3.4 Diagnostic value of indicators based on ATN framework for autoantibody‐negative AE patients

**FIGURE 2:**
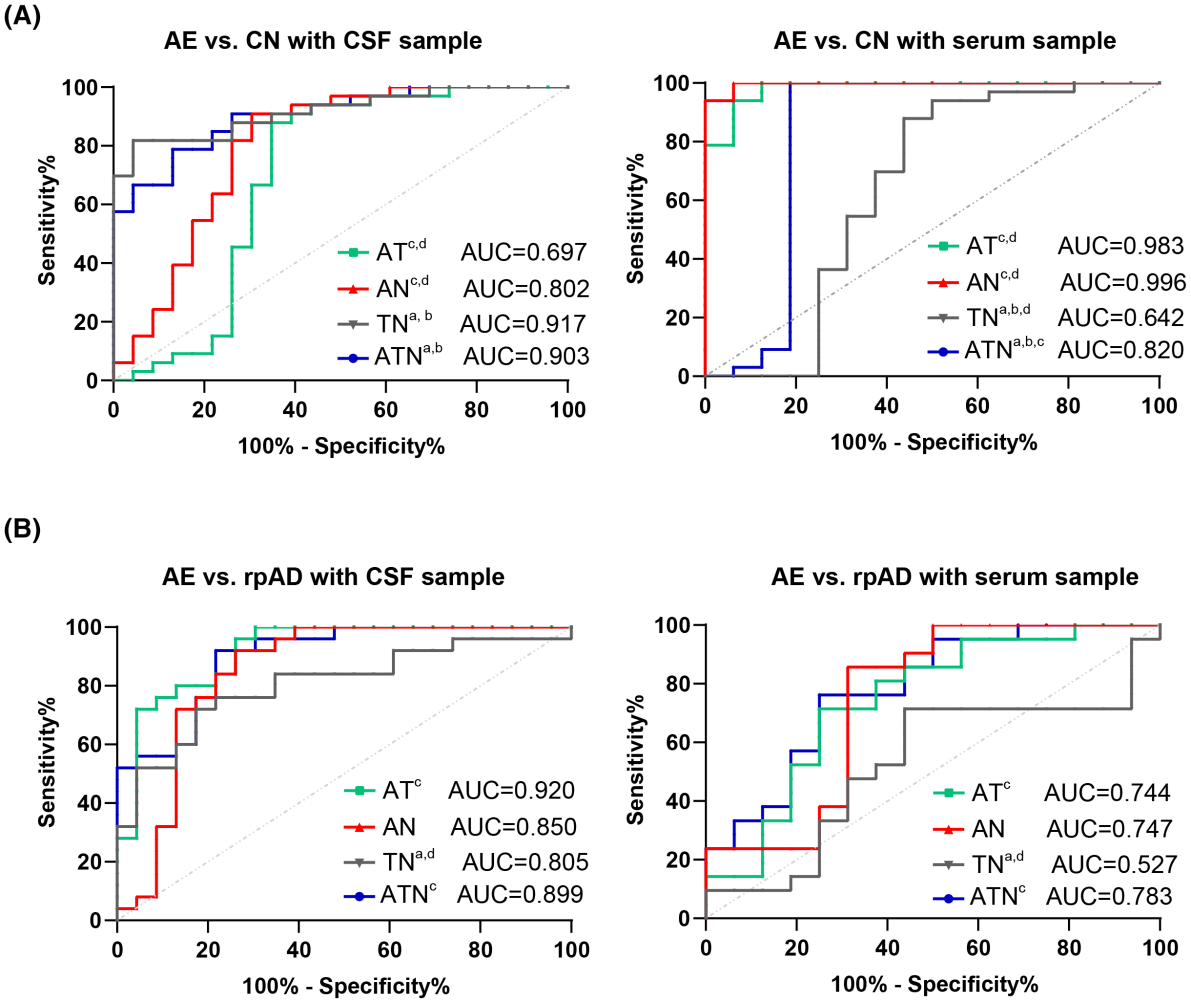
ROC curves of indicators based on ATN framework in distinguishing AE and CN, as well as AE and rpAD. (A) ROC curves of indicators based on ATN framework in distinguishing AE and CN subjects, and comparison of AUCs among *A&T, A&N, T&N*, and *A&T&N*. (B) ROC curves of indicators based on ATN framework in distinguishing AE and CN group, and comparison of AUCs among *A&T, A&N, T&N*, and *A&T&N*. DeLong Test. ^a^ *p* < 0.05 compared to *A&T*, ^b^ *p* < 0.05 compared to *A&N*, ^c^ *p* < 0.05 compared to *T&N*, ^d^ *p* < 0.05 compared to *A&T&N*. AE, autoimmune encephalitis; CN, cognitively normal; CSF, Cerebrospinal fluid; rpAD, rapidly progressive Alzheimer's disease.

**FIGURE 3:**
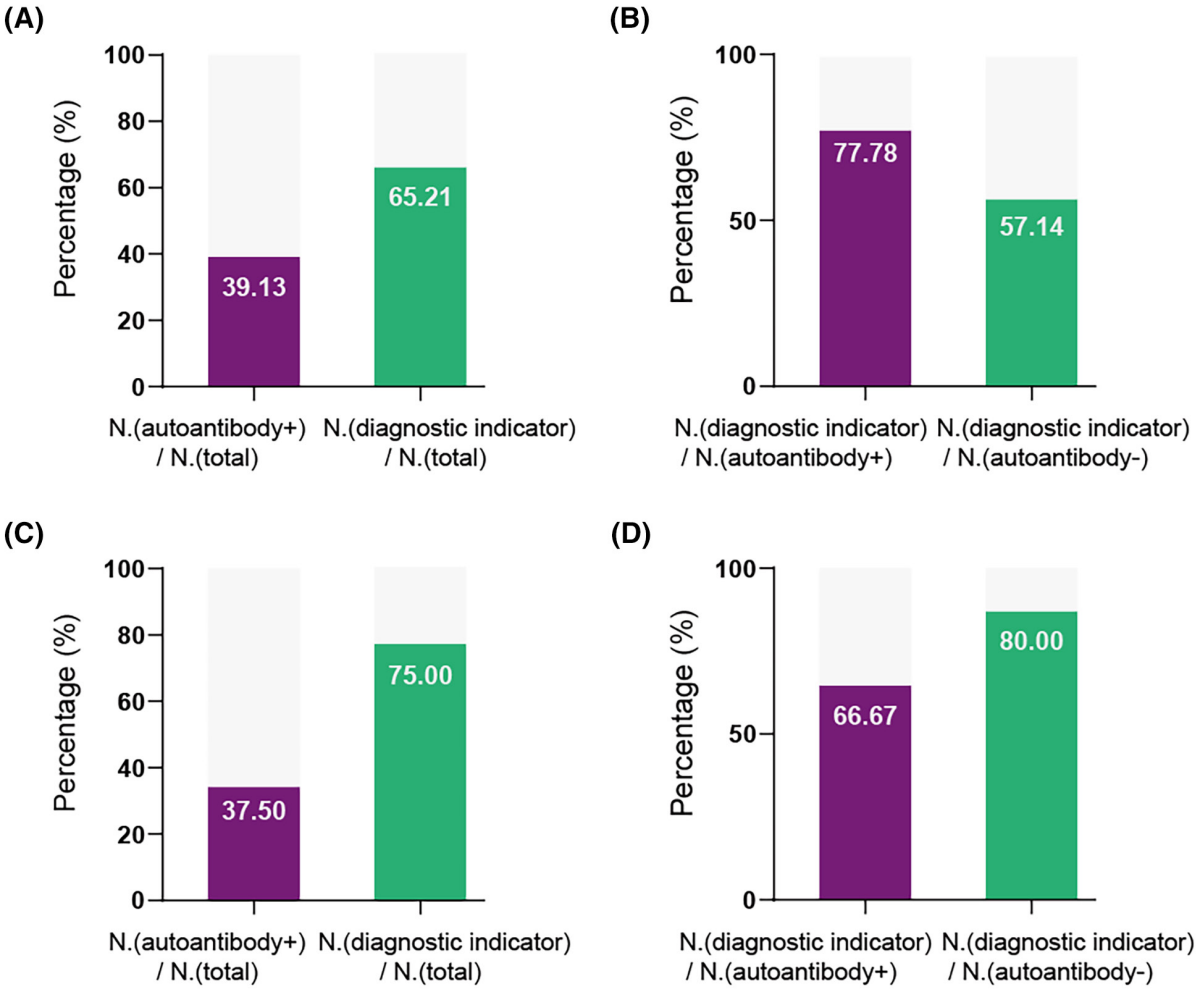
The diagnostic value of indicator based on ATN framework in autoantibody‐negative AE group. (A) Comparison of diagnostic value between autoantibodies and CSF indicator based on ATN framework. (B) Comparison of the percentage of AE patients diagnosed by indicator based on ATN framework using CSF biomarkers between autoantibody‐positive and ‐negative AE groups. (C) Comparison of diagnostic value between autoantibodies and serum indicator based on ATN framework. (D) Comparison of the percentage of AE patients diagnosed by indicator based on ATN framework using serum biomarkers between autoantibody‐positive and ‐negative AE groups. AE, autoimmune encephalitis; ATN, amyloid/tau/neurodegeneration; N. (autoantibody −), numbers of autoantibody‐negative patients.; N. (autoantibody +), numbers of autoantibody‐positive patients; N. (diagnostic indicator), numbers of AE patients diagnosed using indicator based on ATN framework; N. (total), numbers of the whole AE patients.

Clinically suspected antibody‐negative AE cases are difficult to confirm. Therefore, we evaluated the diagnostic power of indicators based on the ATN framework for antibody‐negative AE. First, the diagnostic value of the diagnostic indicator according to the ATN classification was calculated to compare AE to controls and the rpAD group, and a comparison of ROC curves was also made (Figure 2). All the combinations with the ATN framework showed diagnostic value to distinguish AE from controls using CSF samples, while no diagnostic power was shown with *T & N* using serum samples (Figure 2A). All the AUCs for discriminating AE from rpAD using both CSF and serum samples showed diagnostic power (Figure 2B). Then, we evaluated the diagnostic power for distinguishing autoantibody‐negative AE with RPD patients. The number of autoantibodies‐positive individuals only accounted for 39.13% of the total number of AE patients with CSF samples, while the diagnostic positive rate with CSF biomarkers reached 62.51% using indicators with *A&T&N* (Figure 3A). Furthermore, there were 77.78% and 57.14% of subjects who met the criteria using *A&T&N* in the autoantibodies‐positive and –negative individuals, respectively (Figure 3B). Most importantly, The ATN framework also worked when using serum biomarkers. The number of autoantibodies‐positive individuals accounted for 37.50% of the total number of AE patients with serum samples, while the diagnostic positive rate with serum biomarkers reached 75.00% using an indicator of *A&T&N* (Figure 3C). There were 66.67% of subjects who met the criteria using the indicator of *A&T&N* in the autoantibodies‐positive and even 80.00% of subjects in the autoantibodies‐negative individuals (Figure 3D). The Aβ42 and Aβ42/40 were, respectively, included in the diagnostic analysis, and no significant elevation was shown using Aβ42/40 as a marker of amyloid pathology. Only the maximum AUCs were presented here.

### 3.5 Correlations between baseline biomarkers and cognitive decline rate in patients with RPDs

CSF p‐Tau and t‐Tau levels were positively correlated with the rate of cognitive decline in the rpAD group. Besides, CSF Aβ42/p‐Tau negatively correlated with the rate of cognitive decline in the rpAD group but positively correlated with the cognitive decline rate in the AE group (Figure S1A and Table S3). However, after adjustment for age, sex, and follow‐up time, there was only p‐Tau that positively associated with cognitive decline rate in the rpAD group (*R* = 0.512, *p* = 0.018; Figure S1B and Table S3). Serum t‐Tau positively correlated with the cognitive decline rate in the rpAD group after adjustment for age, sex, and follow‐up time (*R* = 0.527, *p* = 0.036; Figure S1C and Table S4). There were no serum biomarkers correlated with the cognitive decline rate in the AE group.

### 3.6 Prognostic value of biomarkers and indicators based on ATN framework at baseline for cognitive decline rate in patients with RPDs

The prognostic power of baseline CSF biomarkers for cognitive decline rate was estimated in patients with RPDs comprising rpAD and AE. (Table S5). Specifically, p‐Tau or t‐Tau alone was able to predict the rate of cognitive decline in rpAD patients, with adjusted *R*‐Square of 0.283 and 0.217, respectively. The prognostic power was not elevated obviously when we used indicators based on the ATN framework in the rpAD group. However, integrative indicators of demographic characteristics with CSF biomarkers performed better in rpAD subjects, particularly, indicators of *ATN & age* showed enhanced prognostic value moderately, with adjusted *R*‐Square 0.391 (*p* = 0.008). Besides, CSF biomarkers had no predictive power for cognitive decline rate in the current AE cohort, and this situation did not change after using the indicators based on the ATN framework. The prognostic value of biomarkers and indicators based on the ATN framework in serum for cognitive decline rate were also evaluated, yet no statistical significance was obtained (Table S6). The Aβ42 and Aβ42/40 were, respectively, included in the prognostic analysis based on the ATN framework, and no elevation was shown using Aβ42/40 as a marker of amyloid pathology here.

### 3.7 Prognostic value of biomarkers and indicators based on ATN framework at baseline for survival in patients with RPDs

The prognostic value of CSF AD biomarkers and indicators based on the ATN framework were estimated for survival outcomes and survival time in the CJD group, as CJD usually progresses rapidly and the lifespan of patients is very poor. CJD patients were divided into a survival group and a death group according to their survival outcomes. We first compared the differences in baseline levels of fluid biomarkers and their ratios between the two groups, and no statistical differences were found (data were not shown). The CSF biomarkers at baseline and indicators based on the ATN framework had no prognostic power for survival outcomes in the CJD group (Table S7). Next, we compared the predictive capability of different CSF biomarkers at the baseline and the indicators based on the ATN framework on the survival time of patients (Table S7). CSF t‐Tau levels were significantly associated with survival time, with an adjusted *R*‐Square of 0.16 (*p* = 0.02). The predictive power was elevated when using the combined indicators including *T&N* and *A&T&N*, with adjusted *R*‐Square of 0.189 (*p* = 0.028), and 0.273 (*p* = 0.014), respectively. Besides, indicators incorporating demographic characteristics containing age and disease duration elevate the prognostic power. The prognostic value of biomarkers and indicators based on the ATN framework in serum for survival were also evaluated, yet no statistical significance was obtained (Table S8). The Aβ42 and Aβ42/40 were, respectively, included in the prognostic analysis, and no elevation was shown using Aβ42/40 as a marker of amyloid pathology here.

## 4 DISCUSSION

The core biomarkers of AD are always strictly related to AD pathophysiology. However, the clinical manifestations of dementia can be attributed to damage to memory‐related circuits or brain regions. Some common pathophysiological changes in AD also appear in other types of dementia, such as neuroinflammation and neuronal necroptosis, therefore, we believe the co‐pathophysiology provides a rationale for applying the ATN framework to other types of dementia. Some studies also applied diagnostic and prognostic models based on the ATN framework to diseases other than AD that can lead to dementia.^22^, ^23^ Hence, the current study explored the diagnostic and prognostic power of indicators based on the ATN framework in three types of RPDs.

A comparison of CSF and serum biomarkers and their rations in AE, CJD, and rpAD was first performed in our study. P‐Tau is considered a specific biomarker for Tau phosphorylation and is related to neurofibrillary tangles, which contribute to AD disease processes.^24^ In this regard, CSF p‐Tau is characteristically elevated in rpAD patients compared to the other two groups. Besides, CSF t‐Tau represents a common biomarker for neuronal degeneration.^25^ Hence, rapid neurodegeneration and paralleling cognitive decline in CJD patients may be associated with high levels of t‐Tau in CSF, as reported in our research. Besides, some researchers reported increased blood t‐Tau in CJD patients than in AD subjects, however, similar results were not obtained here. Different from previous studies, the current study mainly focused on the Chinese Han population. Hence, the discrepancy between the current study and others may be attributed to population and different peripheral metabolic levels. Measuring blood brain‐derived Tau in a multiethnic and multicenter manner may be a promising strategy in the future.^26^ Furthermore, not only to the ratio of CSF Aβ42 and Aβ40, the ratios of Aβ and Tau, as well as t‐Tau and p‐Tau are also reported to be considered as the indicators for diagnosis and prognosis of AD and other types of dementias.^27^, ^28^, ^29^ Therefore, the current study evaluated all these ratios and also extended prior studies by estimating these indicators in blood samples. One of the noteworthy results was that CSF Aβ42/p‐Tau negatively correlated with the rate of cognitive decline in the rpAD group but positively correlated with that in AE group. Indeed, AE has a different pathogenesis from AD. Immune‐mediated antibodies or inflammatory factors attacking neurons are important pathophysiological changes in AE. Neurons initiate a compensatory protection mechanism to face external stress, which may break the original metabolic balance of Aβ42 and p‐Tau. This may explain to a certain extent the opposite results abovementioned. However, after adjustment for age, sex, and follow‐up time, the correlation was missing. A hypothesis‐driven analysis using a large autoantibodies‐confirmed AE cohort may address this question.

Antibody‐negative AE is challenging to diagnose because clinically suspected antibody‐negative AE cases are difficult to confirm using clinical examination.^30^ The current study provides, to our knowledge, the first evidence that the indicators based on the ATN framework had the potential to identify autoantibody‐negative AE patients from individuals with CJD and rpAD. The indicator containing *A&T&N* showed promising diagnostic power using either CSF or serum samples. Therefore, considering ATN framework‐based indicators may be helpful when diagnosing AE in the presence of autoantibodies‐negative, or in a condition where the autoantibodies assay is not available.

The prognostic power of CSF and serum biomarkers and ATN framework‐based indicators were evaluated in the present study. We demonstrated that t‐Tau and p‐Tau in CSF were associated with the rate of cognitive decline in rpAD patients which is in agreement with previous studies,^31^ and on this basis, we developed novel prognostic indicators based on the ATN framework, which had better performance than using CSF biomarkers alone. Furthermore, ATN framework‐based indicators showed better performance for CJD prognosis, specifically, the prognostic power of the *A&T&N‐based* indicator was higher than that of the *T&N‐based* indicator, together with the previous studies that decreased Aβ42 was found in sporadic CJD,^32^, ^33^, ^34^ indicating that CSF Aβ42 may be involved in the progression of CJD. Further explorations involving CSF biomarkers in CJD patients need to consider the impact of Aβ42 levels on the final results. In addition, the ATN framework‐based indicators had no prognostic power using serum biomarkers in the present study, implying that future biomarker research on the prognosis of RPDs should pay more attention to CSF‐based assays.

Recent opinions proposed that adding an “X” factor to the ATN framework‐based indicators could reflect the whole spectrum of AD pathologies.^35^ The X factors represent biomarkers involved in factors including but not limited to neuroinflammation, synaptic damage, and unknown pathologies of AD,^36^ which may also participate in pathologies of CJD and AE. One of the inspirations from the current research is that the combined diagnostic or prognostic indicators of multiple fluid biomarkers always showed better performance. Given that some biomarkers reflecting central nervous system damage including GFAP and NFL have diagnostic or prognostic value for RPDs,^37^, ^38^ future work replicating these findings and optimizing cutoffs will be required before integrating these factors into diagnostic or prognostic indicators.

The current study also has some limitations. First, the neuropathologically verified cases of CJD were lacking in our study and most of the subjects enrolled were clinically probable CJD patients with no 14‐3‐3 protein values, which makes it difficult to compare the diagnostic power between classic biomarker and novel indicators based on ATN framework. Next, current research did not take disease subtypes and genetic factors into account, and results require independent validation in a larger cohort, controlling for potential predictors, such as CJD subtypes, *APOE*‐ε4, and *PRNP* Codon 129 genotype. Then, telephone questionnaires were used to follow up on the cognitive functions of participants in this study, and this follow‐up approach may not be as accurate as face‐to‐face interviews, although telephone‐based questionnaires have been validated.^21^ Last, the current study did not include other types of RPDs (such as vascular, toxic, metabolic, and so on), which will be implemented in future studies.

In conclusion, the current study provided novel perspectives and potential approaches for establishing effective and simple diagnostic and prognostic tools for RPDs including AE, rpAD, and CJD. Further investigations are also needed to validate some of the identified trends in the present study in a broader population.

## AUTHOR CONTRIBUTIONS

J.T.Y. and Q.D. were responsible for the conception and design of the study. Y. C., Y. Y. H., and S. F. C. were responsible for the acquisition of follow‐up data. Y. C., S. F. C., Y. R. Z., K. M. W., Y. Y. H., L. F. Q., K. K., H. Q. L., S. D. C., and W. S. L. were responsible for the acquisition, analysis of data, and preparing the figures and tables. J.T.Y. and Y. C. were responsible for the drafting of the manuscript.

## FUNDING INFORMATION

This study was supported by grants from the Science and Technology Innovation 2030 Major Projects (2022ZD0211600), Shanghai Municipal Science and Technology Major Project (2018SHZDZX01), Research Start‐up Fund of Huashan Hospital (2022QD002), Excellence 2025 Talent Cultivation Program at Fudan University (3030277001), Shanghai Talent Development Funding for The Project (2019074), Shanghai Rising‐Star Program (21QA1408700), and ZHANGJIANG LAB, Tianqiao and Chrissy Chen Institute, the State Key Laboratory of Neurobiology and Frontiers Center for Brain Science of Ministry of Education, Fudan University.

## CONFLICT OF INTEREST STATEMENT

The authors declared no potential conflicts of interest concerning the research, authorship, and/or publication of this article.

## CONSENT TO PARTICIPATE

All participants and their caregivers provided informed consent.

## Supporting information


Data S1.

## Data Availability

The data that support the findings of this study are available from the corresponding author upon reasonable request.
