# Supplementary material for: Novel diagnostic and prognostic approach for rapidly progressive dementias: Indicators based on amyloid/tau/neurodegeneration (ATN) framework
Source: CNS Neurosci Ther. 2024 Jul 16;30(7):e14857. doi: 10.1111/cns.14857 (PMC11251870; doi:10.1111/cns.14857)
Supplement: Supplementary file 1 — Data S1. [file CNS-30-e14857-s001.docx]

**Novel diagnostic and prognostic approach for rapidly progressive dementias: Indicators based on Amyloid/Tau/Neurodegeneration (ATN) framework**

Running title: **ATN** framework **for** diagnosis and prognosis of RPDs

Yuan Cheng ^1,2^, Shu-Fen Chen ^1,2^, Ya-Ru Zhang ^1,2^, Yu Guo ^1,2^, Kai-Min Wu ^1,2^, Yu-Yuan Huang ^1,2^, Qiaolifan Aerqin ^1,2^, Kevin Kuo ^1,2^, Hong-Qi Li ^1,2^, Shi-Dong Chen ^1,2^, Wei-Shi Liu ^1,2^, Qiang Dong ^1,2^, Jin-Tai Yu ^1,2, *^

1. Department of Neurology and Institute of Neurology, Huashan Hospital, State Key Laboratory of Medical Neurobiology and MOE Frontiers Center for Brain Science, Shanghai Medical College, Fudan University, Shanghai, China.

2. National Center for Neurological Disorders, Shanghai, China.

* Correspondance to Dr. Jin-Tai Yu at jintai_yu@fudan.edu.

**Supplementary Figures**

**Figure S1.**


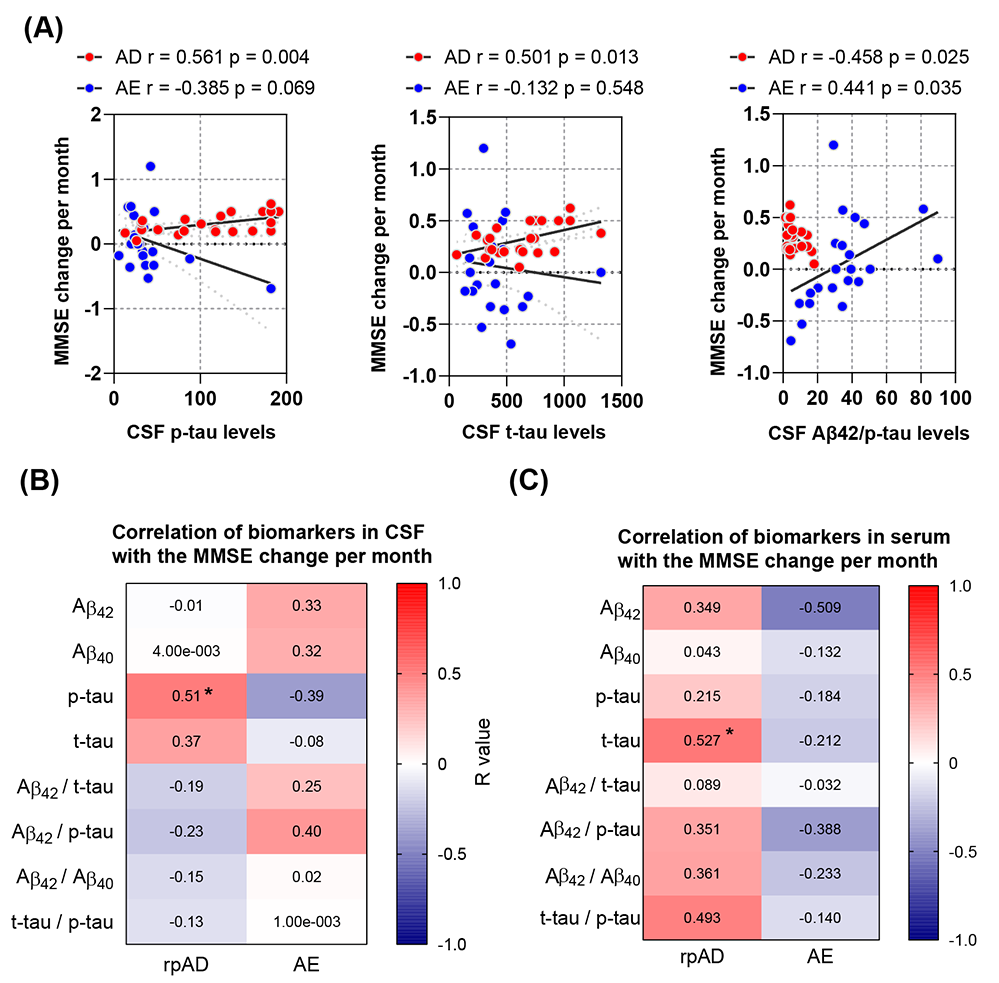


**Figure S1. Correlations between CSF and serum biomarkers at baseline and follow-up cognitive decline rate in patients with RPDs.**

**(A)** The correlations between cognitive decline rate and CSF p-Tau, t-Tau levels, and Aβ42/p-Tau in patients with RPDs comprising rpAD and AE. (**B**) The correlations between cognitive decline rate and CSF biomarkers and their ratios after adjustment for age, sex, and follow-up time in patients with RPDs comprising rpAD and AE. (**C**) The correlations between cognitive decline rate and serum biomarkers and their ratios after adjustment for age, sex, and follow-up time in patients with RPDs comprising rpAD and AE.CSF, Cerebrospinal fluid; Aβ, β-amyloid; p-Tau, phosphorylated Tau protein; t-Tau, total Tau protein; rpAD, rapidly progressive Alzheimer’s disease; AE, autoimmune encephalitis. *P* values are indicated with asterisks: **p* < 0.05.

**Supplementary Tables**

**Table S1. Performance of CSF biomarkers and their ratios to differentiate patients with RPDs.**

| **Biomarkers** | **Aβ42** | | **Aβ40** | | **p-Tau** | | **t-Tau** | |
| --- | --- | --- | --- | --- | --- | --- | --- | --- |
|  | **Cutoff** | AUC (95% CI) | **Cutoff** | AUC (95% CI) | **Cutoff** | AUC (95% CI) | **Cutoff** | AUC (95% CI) |
| rpAD vs. AE | > 560.700 | 0.8348 *  (0.7126 to 0.9570) | < 7497 | 0.6122  (0.4456 to 0.7788) | < 53.800 | 0.8348 *  (0.7132 to 0.9564) | < 695.800 | 0.6974  (0.5453 to 0.8495) |
| CJD vs. rpAD | > 574.500 | 0.7543 *  (0.6216 to 0.8869) | < 4767 | 0.5843  (0.4297 to 0.7389) | < 73.980 | 0.7700 *  (0.6276 to 0.9124) | > 1070.000 | 0.8929 *  (0.7923 to 0.9934) |
| CJD vs. AE | < 685.400 | 0.5683  (0.4096 to 0.7270) | < 5776 | 0.5047  (0.3403 to 0.6690) | > 34.480 | 0.7329 *  (0.5900 to 0.8759) | > 752.300 | 0.9037 *  (0.8117 to 0.9957) |
| **Biomarker ratios** | **Aβ42 / p-Tau** | | **Aβ42 / t-Tau** | | **Aβ42 / Aβ40** | | **t-Tau / p-Tau** | |
|  | **Cutoff** | AUC (95% CI) | **Cutoff** | AUC (95% CI) | **Cutoff** | AUC (95% CI) | **Cutoff** | AUC (95% CI) |
| rpAD vs. AE | > 14.750 | 0.9443 *  (0.8860 to 1.0000) | > 1.515 | 0.8435 *  (0.7242 to 0.9628) | > 0.095 | 0.9139 *  (0.8297 to 0.9982) | > 5.945 | 0.8139 *  (0.6866 to 0.9412) |
| CJD vs. rpAD | > 10.770 | 0.8714 *  (0.7754 to 0.9674) | < 0.600 | 0.6164  (0.4637 to 0.7691) | > 0.095 | 0.8436 *  (0.7251 to 0.9621) | > 10.960 | 0.9686 *  (0.9290 to 1.000) |
| CJD vs. AE | < 27.580 | 0.7329 *  (0.5846 to 0.8812) | < 1.511 | 0.8727 *  (0.7755 to 0.9698) | < 0.1833 | 0.5280  (0.3647 to 0.6912) | > 10.90 | 0.8152 *  (0.6899 to 0.9405) |

Abbreviations: AUC, Area under the curve; Aβ, β-amyloid; p-Tau, phosphorylated Tau protein; t-Tau, total Tau protein. *P* values are indicated with asterisks: *p* < 0.05.

**Table S2. Performance of serum biomarkers and their ratios to differentiate patients with RPDs.**

| **Biomarkers** | **Aβ42** | | **Aβ40** | | **p-Tau** | | **t-Tau** | |
| --- | --- | --- | --- | --- | --- | --- | --- | --- |
|  | **Cutoff** | **AUC (95% CI)** | **Cutoff** | **AUC (95% CI)** | **Cutoff** | **AUC (95% CI)** | **Cutoff** | **AUC (95% CI)** |
| rpAD vs. AE | > 10.06 | 0.7778 *  (0.6225 to 0.9331) | > 559.3 | 0.5188  (0.3172 to 0.7203) | > 9.70 | 0.5193  (0.3244 to 0.7143) | > 69.69 | 0.5030  (0.3129 to 0.6931) |
| CJD vs. rpAD | > 9.10 | 0.6655  (0.4944 to 0.8365) | > 473.6 | 0.5132  (0.3249 to 0.7014) | > 9.30 | 0.7744 *  (0.6314 to 0.9173) | > 151.20 | 0.6179  (0.4441 to 0.7917) |
| CJD vs. AE | < 18.57 | 0.5917  (0.3990 to 0.7844) | < 557.1 | 0.5181  (0.3192 to 0.7170) | > 11.40 | 0.7902 *  (0.6451 to 0.9353) | > 153.20 | 0.6354  (0.4550 to 0.8158) |
| **Biomarkers** | **Aβ42 / p-Tau** | | **Aβ42 / t-Tau** | | **Aβ42 / Aβ40** | | **t-Tau / p-Tau** | |
|  | **Cutoff** | **AUC (95% CI)** | **Cutoff** | **AUC (95% CI)** | **Cutoff** | **AUC (95% CI)** | **Cutoff** | **AUC (95% CI)** |
| rpAD vs. AE | > 1.368 | 0.8159 *  (0.6760 to 0.9558) | > 0.128 | 0.8222 *  (0.6776 to 0.9669) | > 0.048 | 0.7567 *  (0.5963 to 0.9171)) | > 43.38 | 0.5179  (0.3243 to 0.7114) |
| CJD vs. rpAD | > 0.604 | 0.5726  (0.3943 to 0.7509) | > 0.064 | 0.6012  (0.4244 to 0.7780) | > 0.020 | 0.6750  (0.5026 to 0.8474) | < 19.26 | 0.5272  (0.3484 to 0.7061) |
| CJD vs. AE | < 2.072 | 0.7917 *  (0.6323 to 0.9510) | < 0.200 | 0.7483 *  (0.5761 to 0.9206) | < 0.026 | 0.5389  (0.3372 to 0.7406) | < 31.57 | 0.5223  (0.3289 to 0.7157) |

Abbreviations: AUC, Area under the curve; Aβ, β-amyloid; p-Tau, phosphorylated Tau protein; t-Tau, total Tau protein. *P* values are indicated with asterisks: *p* < 0.05.

**Table S3. Correlations between CSF biomarkers at baseline and the average change of MMSE score per month in RPDs due to rpAD and AE.**

| **rpAD group** | | | | | | | | | |
| --- | --- | --- | --- | --- | --- | --- | --- | --- | --- |
|  | Aβ42 | Aβ40 | p-tau | t-tau | | Aβ42 / t-tau | Aβ42 / p-tau | Aβ42 / Aβ40 | t-tau / p-tau |
| R | 0.229 | 0.177 | 0.561 | 0.501 | | -0.316 | -0.458 | -0.117 | -0.301 |
| *P* | 0.281 | 0.408 | 0.004* | 0.013* | | 0.132 | 0.025* | 0.586 | 0.153 |
| (adjusting for age、sex and follow-up duration) | | | | | | | | | |
| R’ | -0.012 | 0.004 | 0.512 | 0.365 | -0.192 | | -0.228 | -0.147 | -0.125 |
| *P’* | 0.958 | 0.987 | 0.018* | 0.104 | 0.403 | | 0.320 | 0.526 | 0.590 |
| **AE group** | | | | | | | | | |
|  | Aβ42 | Aβ40 | p-tau | t-tau | Aβ42 / t-tau | | Aβ42 / p-tau | Aβ42 / Aβ40 | t-tau / p-tau |
| R | 0.357 | 0.185 | -0.385 | -0.132 | 0.331 | | 0.441 | 0.312 | -0.012 |
| *P* | 0.094 | 0.398 | 0.069 | 0.548 | 0.123 | | 0.035* | 0.147 | 0.952 |
| (adjusting for age、sex and follow-up duration) | | | | | | | | | |
| R’ | 0.334 | 0.315 | -0.388 | -0.083 | 0.249 | | 0.404 | 0.018 | 0.001 |
| *P’* | 0.150 | 0.176 | 0.091 | 0.729 | 0.291 | | 0.077 | 0.448 | 0.997 |

Abbreviations: Aβ, β-amyloid; p-Tau, phosphorylated Tau protein; t-Tau, total Tau protein; rpAD, rapidly progressive Alzheimer’s disease; AE, autoimmune encephalitis; AT, Amyloid / Tau; AN, Amyloid / Neurodegeneration; TN, Tau / Neurodegeneration; ATN, Amyloid / Tau / Neurodegeneration. The bold font indicates statistical differences. *P* values are indicated with asterisks: *p* < 0.05.

**Table S4. Correlations between serum biomarkers at baseline and the average change of MMSE score per month in RPDs due to rpAD and AE.**

| **rpAD group** | | | | | | | | | |
| --- | --- | --- | --- | --- | --- | --- | --- | --- | --- |
|  | Aβ42 | Aβ40 | p-tau | t-tau | | Aβ42 / t-tau | Aβ42 / p-tau | Aβ42 / Aβ40 | t-tau / p-tau |
| R | 0.292 | -0.076 | 0.105 | 0.374 | | 0.102 | 0.214 | 0.372 | 0.109 |
| *P* | 0.212 | 0.750 | 0.660 | 0.104 | | 0.669 | 0.364 | 0.116 | 0.646 |
| (adjusting for age、sex and follow-up time) | | | | | | | | | |
| R’ | 0.349 | 0.043 | 0.215 | 0.527 | 0.089 | | 0.351 | 0.361 | 0.493 |
| *P’* | 0.186 | 0.875 | 0.424 | 0.036* | 0.718 | | 0.183 | 0.169 | 0.052 |
| **AE group** | | | | | | | | | |
|  | Aβ42 | Aβ40 | p-tau | t-tau | Aβ42 / t-tau | | Aβ42 / p-tau | Aβ42 / Aβ40 | t-tau / p-tau |
| R | -0.303 | -0.565 | -0.018 | -0.400 | -0.061 | | -0.211 | -0.336 | -0.362 |
| *P* | 0.254 | 0.837 | 0.948 | 0.125 | 0.830 | | 0.451 | 0.211 | 0.196 |
| (adjusting for age、sex and follow-up time) | | | | | | | | | |
| R’ | -0.509 | -0.132 | -0.184 | -0.212 | -0.032 | | -0.388 | -0.233 | -0.140 |
| *P’* | 0.091 | 0.683 | 0.568 | 0.509 | 0.921 | | 0.213 | 0.485 | 0.664 |

Abbreviations: Aβ, β-amyloid; p-Tau, phosphorylated Tau protein; t-Tau, total Tau protein; rpAD, rapidly progressive Alzheimer’s disease; AE, autoimmune encephalitis; AT, Amyloid / Tau; AN, Amyloid / Neurodegeneration; TN, Tau / Neurodegeneration; ATN, Amyloid / Tau / Neurodegeneration. The bold font indicates statistical differences. *P* values are indicated with asterisks: *p* < 0.05.

**Table S5. Prognostic value of CSF indicators based on ATN framework at baseline for cognitive decline rate in patients with RPDs comprising rpAD and AE.**

| Group | Biomarkers / Indicators | R | R-Square | Adjusted R-Square | P value |
| --- | --- | --- | --- | --- | --- |
| **rpAD** | Aβ42 | 0.229 | 0.053 | 0.010 | 0.281 |
|  | Aβ40 | 0.177 | 0.031 | -0.013 | 0.408 |
|  | p-Tau | 0.561 | 0.314 | 0.283 | 0.004 * |
|  | t-Tau | 0.510 | 0.251 | 0.217 | 0.013 * |
|  | AT | 0.561 | 0.315 | 0.249 | 0.019 |
|  | AN | 0.501 | 0.251 | 0.179 | 0.048 |
|  | TN | 0.584 | 0.341 | 0.278 | 0.013 |
|  | ATN | 0.587 | 0.344 | 0.246 | 0.035 |
|  | ATN & age | 0.705 | 0.497 | 0.391 | 0.008 * |
|  | ATN & duration | 0.590 | 0.348 | 0.210 | 0.074 |
|  | | | | | |
| **AE** | Aβ42 | 0.357 | 0.128 | 0.086 | 0.094 |
|  | Aβ40 | 0.185 | 0.034 | -0.012 | 0.398 |
|  | p-Tau | 0.385 | 0.149 | 0.108 | 0.069 |
|  | t-Tau | 0.132 | 0.017 | -0.029 | 0.548 |
|  | AT | 0.537 | 0.289 | 0.218 | 0.063 |
|  | AN | 0.376 | 0.141 | 0.055 | 0.218 |
|  | TN | 0.396 | 0.157 | 0.073 | 0.181 |
|  | ATN | 0.543 | 0.294 | 0.183 | 0.079 |
|  | AT & age | 0.540 | 0.291 | 0.179 | 0.082 |
|  | AT & duration | 0.546 | 0.298 | 0.187 | 0.076 |

Abbreviations: Aβ, β-amyloid; p-Tau, phosphorylated Tau protein; t-Tau, total Tau protein; rpAD, rapidly progressive Alzheimer’s disease; AE, autoimmune encephalitis; AT, Amyloid / Tau; AN, Amyloid / Neurodegeneration; TN, Tau / Neurodegeneration; ATN, Amyloid / Tau / Neurodegeneration. *P* values are indicated with asterisks: *p* < 0.05.

| **Group** | **Biomarkers / Indicators** | **R** | **R-Square** | **Adjusted R-Square** | ***P* value** |
| --- | --- | --- | --- | --- | --- |
| **rpAD** | Aβ42 | 0.425 | 0.180 | 0.135 | 0.062 |
|  | Aβ40 | 0.076 | 0.006 | -0.050 | 0.752 |
|  | p-Tau | 0.283 | 0.080 | 0.029 | 0.226 |
|  | t-Tau | 0.434 | 0.189 | 0.143 | 0.056 |
|  | AT | 0.444 | 0.197 | 0.103 | 0.155 |
|  | AN | 0.580 | 0.258 | 0.170 | 0.079 |
|  | TN | 0.493 | 0.243 | 0.153 | 0.094 |
|  | ATN | 0.528 | 0.279 | 0.143 | 0.146 |
|  | | | | | |
| **AE** | Aβ42 | 0.400 | 0.160 | 0.100 | 0.124 |
|  | Aβ40 | 0.059 | 0.004 | -0.068 | 0.827 |
|  | p-Tau | 0.240 | 0.057 | -0.010 | 0.371 |
|  | t-Tau | 0.247 | 0.061 | -0.006 | 0.356 |
|  | AT | 0.400 | 0.160 | 0.031 | 0.321 |
|  | AN | 0.376 | 0.141 | 0.055 | 0.218 |
|  | TN | 0.355 | 0.126 | 0.009 | 0.418 |
|  | ATN | 0.451 | 0.204 | 0.005 | 0.416 |

**Table S6. Prognostic value of serum indicators based on ATN framework at baseline for cognitive decline rate in patients with RPDs comprising rpAD and AE.**

Abbreviations: Aβ, β-amyloid; p-Tau, phosphorylated Tau protein; t-Tau, total Tau protein; rpAD, rapidly progressive Alzheimer’s disease; AE, autoimmune encephalitis; AT, Amyloid / Tau; AN, Amyloid / Neurodegeneration; TN, Tau / Neurodegeneration; ATN, Amyloid / Tau / Neurodegeneration. *P* values are indicated with asterisks: *p* < 0.05.

**Table S7 Prognostic value of CSF biomarkers and indicators based on ATN framework at baseline for survival outcomes and survival time in patients with RPD consisting of CJD.**

| **Model** | **Biomarkers / Indicators** | **B (SE)** | **Exp (B)** | | **95% CI** | ***P* value** |
| --- | --- | --- | --- | --- | --- | --- |
| **Model 1**  **(Logistic regression)** | AT (p-Tau) | 0.005 (0.019) | 1.005 | | 0.969 to 1.043 | 0.782 |
|  | AN (Aβ42) | 0.001 (0.001) | 1.001 | | 1.000 to 1.003 | 0.082 |
|  | AN (t-Tau) | -0.001 (0.001) | 0.999 | | 0.998 to 1.001 | 0.459 |
|  | TN ( p-Tau) | 0.022 (0.017) | 1.019 | | 0.989 to 1.056 | 0.188 |
|  | TN ( t-Tau) | -0.001 (0.001) | 0.999 | | 0.997 to 1.001 | 0.389 |
|  | ATN ( Aβ42) | 0.001 (0.001) | 1.001 | | 0.999 to 1.003 | 0.212 |
|  | ATN ( p-Tau ) | 0.010 (0.019) | 1.010 | | 0.972 to 1.049 | 0.606 |
|  | ATN ( t-Tau ) | -0.001 (0.001) | 0.999 | | 0.997 to 1.001 | 0.391 |
|  | | | | | | |
| **Model 2**  **(Linear regression)** | **Biomarkers / Indicators** | **R** | **R-Square** | **Adjusted R-Square** | | ***P* value** |
|  | Aβ42 | 0.208 | 0.043 | 0.006 | | 0.288 |
|  | p-Tau | 0.086 | 0.007 | -0.031 | | 0.662 |
|  | t-Tau | 0.437 | 0.191 | 0.160 | | 0.020 * |
|  | AT | 0.322 | 0.104 | 0.032 | | 0.255 |
|  | AN | 0.459 | 0.211 | 0.148 | | 0.052 |
|  | TN | 0.499 | 0.249 | 0.189 | | 0.028 * |

|  | ATN | 0.595 | 0.354 | 0.273 | 0.014 * |
| --- | --- | --- | --- | --- | --- |

|  | ATN & age | 0.596 | 0.355 | 0.243 | 0.033 * |
| --- | --- | --- | --- | --- | --- |

|  | ATN & duration | 0.604 | 0.365 | 0.255 | 0.028 * |
| --- | --- | --- | --- | --- | --- |

Abbreviations: Aβ, β-amyloid; p-Tau, phosphorylated Tau protein; t-Tau, total Tau protein; CJD, Creutzfeldt-Jakob disease; AT, Amyloid / Tau; AN, Amyloid / Neurodegeneration; TN, Tau / Neurodegeneration; ATN, Amyloid / Tau / Neurodegeneration. *P* values are indicated with asterisks: *p* < 0.05.

**Table S8 Prognostic value of serum biomarkers at baseline and indicators based on ATN framework for survival outcomes and survival time in patients with RPD consisting of CJD.**

| **Model** | **Biomarkers / Indicators** | **B (SE)** | **Exp (B)** | | ***P* value** |
| --- | --- | --- | --- | --- | --- |
| **Model 1**  **(logistic regression)** | AT (Aβ42) | -0.105 (0.058) | 0.901 | | 0.072 |
|  | AT (p-tau) | 1.596 (0.902) | 4.931 | | 0.077 |
|  | AN (Aβ42) | -0.084 (0.048) | 0.919 | | 0.082 |
|  | AN (t-tau) | 0.007 (0.004) | 1.007 | | 0.094 |
|  | TN ( p-tau) | 0.462 (0.584) | 1.587 | | 0.429 |
|  | TN ( t-tau) | 0.000 (0.003) | 1.000 | | 0.902 |
|  | ATN ( Aβ42) | -0.120 (0.062) | 0.887 | | 0.154 |
|  | ATN ( p-tau ) | 1.335 (0.983) | 3.799 | | 0.174 |
|  | ATN ( t-tau ) | 0.003 (0.004) | 1.003 | | 0.395 |
|  | | | | | |
| **Model 2**  **(Linear regression)** | **Biomarkers / Indicators** | **R** | **R-Square** | **Adjusted R-Square** | ***P* value** |
|  | Aβ42 | 0.293 | 0.086 | 0.038 | 0.198 |
|  | p-tau | 0.241 | 0.058 | 0.009 | 0.292 |
|  | t-tau | 0.379 | 0.144 | 0.099 | 0.090 |
|  | AT | 0.306 | 0.093 | -0.007 | 0.414 |
|  | AN | 0.387 | 0.150 | 0.055 | 0.233 |
|  | TN | 0.381 | 0.145 | 0.050 | 0.244 |
|  | ATN | 0.391 | 0.153 | 0.003 | 0.408 |

Abbreviations: Aβ, β-amyloid; p-Tau, phosphorylated Tau protein; t-Tau, total Tau protein; CJD, Creutzfeldt-Jakob disease; AT, Amyloid / Tau; AN, Amyloid / Neurodegeneration; TN, Tau / Neurodegeneration; ATN, Amyloid / Tau / Neurodegeneration. *P* values are indicated with asterisks: *p* < 0.05.
